# Impact of Subdomains of Affective and Cognitive Empathy on Burnout Syndrome in Nurses: A Meta‐Analysis

**DOI:** 10.1111/inr.70173

**Published:** 2026-03-19

**Authors:** Madson Alan Maximiano‐Barreto, Adrieli Oliveira Raminelli, Bruna Moretti Luchesi, Marcos Hortes Nisihara Chagas, Marisa Matias, Flávia de Lima Osório

**Affiliations:** ^1^ Department of Neuroscience and Behavioral Sciences University of São Paulo Ribeirão Preto São Paulo Brazil; ^2^ Research Group on Mental Health, Cognition and Aging University of São Paulo Ribeirão Preto São Paulo Brazil; ^3^ Integrated Health Institute, Federal University of Mato Grosso do Sul Campo Grande Mato Grosso do Sul Brazil; ^4^ School of Psychology and Educational Sciences, Center for Psychology University of Porto Porto Portugal; ^5^ National Institute of Science and Technology (INCT‐TM, CNPq) Brasília Brazil

**Keywords:** affective empathy, burnout syndrome, cognitive empathy, empathy, meta‐analyses, nursing

## Abstract

**Aim:**

To investigate the association between empathy and the subdomains of affective and cognitive empathy and burnout syndrome in nurses from any health field.

**Background:**

Empathy is an ability composed of affective and cognitive domains that have been widely studied in healthcare providers. Affective and cognitive empathy have subdomains, and the associations with burnout syndrome are under‐investigated. Burnout syndrome is an occupational disease that often affects nurses.

**Methods:**

A meta‐analysis was conducted guided by the PRISMA Statement checklist and was registered in the PROSPERO database (CRD42024600740). Twenty‐nine studies met the eligibility criteria.

**Results:**

A negative correlation was found between global empathy and burnout syndrome. When considering the subdomains of affective empathy, no statistically significant correlation was found with empathic concern, whereas a moderate positive correlation was found between personal distress and burnout syndrome. Considering the subdomains of cognitive empathy, a weak negative correlation was found between perspective taking and burnout syndrome, whereas a weak positive correlation with fantasy was found.

**Discussion:**

The subdomains of empathy are distinctly related to burnout syndrome in nurses. Perspective taking served as a protective factor for the mental health of these health professionals.

**Conclusions:**

Global empathy was negatively associated with burnout syndrome, while its subdomains showed distinct and sometimes opposing associations. When considering the subdomains of affective and cognitive empathy, distinct interactions were identified. Personal distress and fantasy were positively correlated with burnout syndrome, whereas perspective taking was negatively correlated.

**Implications for nursing and health policy:**

Understanding the impact of empathy subdomains can assist in the planning of interventions aimed at optimizing empathic functioning while minimizing risks to nurses’ mental health.

## 1 Introduction

Empathy is a multidimensional ability that involves behavioral, cognitive, and emotional changes in those who experience it (Davis 1980, 1983; Eisenberg and Strayer 1990; Mercer and Reynolds 2002). This ability is divided into two main domains (affective and cognitive empathy) (Davis 1980). The affective domain is related to the ability of individuals (e.g., nurses) to put themselves in the place of others (e.g., patients) and experience the same feelings and emotions, whether positive or negative. The cognitive domain enables the empathic individual (e.g., nurse) to understand the situation of others (e.g., patients) without taking on their emotions and feelings (Davis 1980).

The main domains of empathy (affective and cognitive) are divided into subdomains (Davis 1980). Affective empathy is composed of empathic concern (i.e., enabling care and concern for the well‐being of the target subject) and personal distress (i.e., enabling the empathizing individual to experience the emotions and feelings of the target subject). Unlike empathic concern, personal distress is related to the negative aspects experienced by the target subject (Davis 1980, 1983). The cognitive domain of empathy comprises perspective taking (i.e., ability to rationally and consciously understand another's point of view) and fantasy (i.e., ability to imagine oneself experiencing situations experienced by the target subject) (Davis 1980, 1983). It is noteworthy that this latter subdomain of cognitive empathy (i.e., fantasy) is measured exclusively by the Interpersonal Reactivity Index (Davis 1983), which has been widely used across diverse samples (Maximiano‐Barreto et al. 2020). Nevertheless, the theoretical status of fantasy remains controversial despite its widespread use, with both classical (e.g., Koller and Camino 2001) and contemporary authors (e.g., Mahmoudi et al. 2022; Shiota and Nomura 2022), suggesting its exclusion from the empathy construct. However, its inclusion in the present meta‐analysis is justified on methodological and comparative grounds, allowing for a critical evaluation of its role in burnout syndrome.

Empathy has been investigated in different contexts, mainly in individuals who provide care (e.g., physicians, students, caregivers of older people, and nurses) (Maximiano‐Barreto et al. 2020; Maximiano‐Barreto et al. 2024a; Taleghani et al. 2017; Wercelens et al. 2023). Empathy is related to humanized practice (Babaii et al. 2021; Suazo et al. 2020). Healthcare providers with high levels of this ability communicate better with patients (Derksen et al. 2013), provide a welcoming space, and reduce fear levels in patients (Babaii et al. 2021). Although higher levels of empathy can be positive for patients, conversely, higher levels of this ability can negatively impact caregivers, resulting in psychological concerns (e.g., anxiety, depression, and burnout syndrome) (Cairns et al. 2024; Maximiano‐Barreto et al. 2024a). However, a meta‐analysis found that global empathy was not associated with depressive symptoms (Yan et al. 2021), which demonstrates the need to continue investigating the relationships between this construct and psychological concerns. It is also necessary to assess the subdomains of affective and cognitive empathy, which have been under‐investigated.

In the healthcare context, empathy is directly linked to the concept of humanized care (Suazo et al. 2020), commonly understood as a set of practices that value different dimensions (e.g., ethics, communication, care) (Cruz Riveros 2020). In this regard, nurses play an important role in humanized care, given that these professionals maintain close and continuous contact with patients (WHO 2020). Empathy is frequently described as one of the primary psychological mechanisms through which humanized care is operationalized in nursing practice (e.g., quality of communication, therapeutic relationships) (Meneses‐La‐Riva et al. 2021; Prieto‐de Benito et al. 2025). Because empathy constitutes a fundamental component of humanized nursing care, it may also have significant implications for these professionals' mental health (Prieto‐de Benito et al. 2025). The diverse demands placed on nurses raise important questions regarding how different dimensions of empathy may function as protective factors or risk factors for adverse psychological outcomes, such as burnout syndrome. Understanding the association between the various components of empathy is essential for informing nursing practices and interventions aimed at preserving nurses' mental health and ensuring the quality of patient care.

Higher levels of affective empathy can exert a direct impact on psychological concerns (see Cairns et al. 2024; Delgado et al. 2023; Maximiano‐Barreto et al. 2024a; Schreiter et al. 2013), whereas higher levels of cognitive empathy can minimize the impact (see Cairns et al. 2024; Maximiano‐Barreto et al. 2024a; Delgado et al. 2023). Moreover, some sociodemographic characteristics (e.g., an advanced age, being female, being married, and having children) identified among health professionals and students with higher levels of global empathy (Maximiano‐Barreto et al. 2020) are also found in nurses with high levels of burnout (Membrive‐Jiménez et al. 2020).

Burnout syndrome was first described in the 1970s (Freudenberger 1974) and was listed as an occupational disease in 2019 with the release of the 11th edition of the International Classification of Diseases (ICD‐11) (WHO 2019). Prolonged exposure to stressors in the workplace (e.g., heavy workload, lack of resources for conducting activities, etc.) increases the likelihood of the development of burnout syndrome, favoring the occurrence of physical and psychological impacts (Maslach and Goldberg 1998; Maslach et al. 2001). Depersonalization, emotional exhaustion, and a lack of personal fulfillment are the main components of this syndrome (Maslach and Goldberg 1998; Maslach et al. 2001).

Burnout syndrome can occur among workers in different occupations (Leiter and Schaufeli 1996). However, nurses are the most affected (Galanis et al. 2023). A meta‐analysis found an 11.23% overall frequency of burnout syndrome in nursing staff of different specialties (i.e., mental health, pediatrics, oncology, etc.) (Woo et al. 2020). During the COVID‐19 pandemic, the frequency of burnout syndrome reached 45%, which was a 34% increase compared with pre‐pandemic years (Nagarajan et al. 2024).

Just as the COVID‐19 pandemic was considered a factor that increased the prevalence of burnout syndrome among nurses, other factors already established in the literature contribute to the occurrence of this occupational disease (e.g., role ambiguity, workload, professional devaluation, etc.) (Duquette et al. 1994; Dall'Ora et al. 2020), generating impacts on the physical and psychological health and, consequently, the quality of life of these professionals (Khatatbeh et al. 2022). The presence of this syndrome among healthcare providers can have direct consequences for patients (e.g., medication errors, poor quality of care, etc.) (Dall'Ora et al. 2020). Empathy appears to be another factor, which can be both a risk factor for burnout syndrome and a protective factor, depending on the domain assessed, as identified in systematic reviews and recent meta‐analyses of studies involving caregivers of older people (Maximiano‐Barreto et al. 2024a), medical students (Cairns et al. 2024). This association has been observed among healthcare professionals (Delgado et al. 2023; Martingano et al. 2025) as well as among non‐professionals (Martingano et al. 2025; Schreiter et al. 2013). However, it remains unclear how the subdomains of empathy are associated with burnout syndrome among nurses.

As described previously, affective empathy and cognitive empathy show distinct interactions with burnout syndrome (see Cairns et al. 2024; Delgado et al. 2023; Maximiano‐Barreto et al. 2024a). These distinct associations between the variables may be related to the different functional roles of empathy subdomains, which remain underexplored in the literature. The cognitive domain of empathy has been associated with emotion regulation capacities, enabling individuals to understand others’ experiences while maintaining psychological distance, whereas affective empathy is less strongly linked to regulatory processes (Thompson et al. 2022). In addition, compassion fatigue may also help explain these divergent patterns, as evidence suggests a negative association with cognitive (e.g., perspective taking; see Morawetz et al. 2022, 2020) empathy and a positive association with affective empathy (Shen et al. 2024), particularly forms characterized by personal distress (Zhang et al. 2021). These factors may help to explain why cognitive empathy tends to function as a protective factor against burnout syndrome, whereas affective empathy may increase vulnerability under conditions of emotional demand among healthcare professionals (e.g., nurses).

Empathy has been directly associated with burnout syndrome among nurses (Hunt et al. 2017) in different specialties, such as mental health (Román‐Sánchez et al. 2022), urgent and emergency care (Viana and Kawagoe 2023), oncology (Taleghani et al. 2017), and others (Hunt et al. 2017). The direct relationship between these variables in nurses was previously reported in a systematic review (Hunt et al. 2017). However, this review had limitations, such as not including the nursing specialties of mental health, pediatrics, and neonatology and not differentiating the subdomains of affective empathy (i.e., empathic concern and personal distress) and cognitive empathy (i.e., perspective taking and fantasy). This last aspect is of the utmost importance, as studies have demonstrated that different regions of the brain are activated when an individual experiences affective and cognitive empathy (Decety et al. 2016; Derntl et al. 2010; Shamay‐Tsoory et al. 2011; Shamay‐Tsoory et al. 2013) and these domains are associated with psychological concerns (e.g., burnout syndrome) in distinct ways (Cairns et al. 2024; Delgado et al. 2023; Maximiano‐Barreto et al. 2024a). A recent meta‐analysis addressed the relationship between the subdomains of affective and cognitive empathy and psychological concerns (e.g., burnout syndrome) in unpaid caregivers and found that the domains and subdomains of empathy have different associations with burnout syndrome (Huo et al. 2025).

Broadening knowledge on the relationship between the subdomains of affective and cognitive empathy and burnout syndrome among nurses can assist in the planning of interventions aimed at mitigating the impacts of this syndrome. However, as mentioned above, literature reviews have focused on relationships between global empathy (Hunt et al. 2017) and its domains (Cairns et al. 2024; Delgado et al. 2023; Maximiano‐Barreto et al. 2024a) and burnout syndrome. The relationship between the subdomains of affective and cognitive empathy and psychological concerns, including burnout syndrome, was recently investigated in meta‐analyses of unpaid caregivers of older people (Huo et al. 2025) and health care professionals (Delgado et al. 2023). However, the authors restricted their analyses to the subdomains of perspective taking and empathic concern, leaving other theoretically relevant subdomains unexamined and focusing on populations whose caregiving contexts differ substantially from professional nursing practice. Despite the relevance of these syntheses, evidence remains fragmented with regard to nurses as a distinct professional group, whose work is characterized by sustained emotional engagement, high workload, and close patient contact across diverse clinical settings. Therefore, by focusing exclusively on nurses and systematically disentangling global empathy from all major affective (i.e., empathic concern and personal distress) and cognitive (i.e., perspective taking and fantasy) subdomains, the aim of the present meta‐analysis was to investigate relationships between global empathy as well as the subdomains of affective and cognitive empathy and burnout syndrome in nurses across all fields. Considering that affective and cognitive empathy involve distinct psychological processes that may function as risk or protective factors for burnout syndrome, the following hypotheses were formulated:

- Higher levels of global empathy significantly correlate with higher levels of burnout syndrome in nurses, as identified in reviews involving healthcare providers (see Hunt et al. 2017; Maximiano‐Barreto et al. 2024c);
- Higher levels of empathic concern and personal distress (subdomains of affective empathy) correlate positively with burnout syndrome, whereas higher levels of perspective taking and fantasy (subdomains of cognitive empathy) correlate negatively with burnout syndrome.

## 2 Methods

### 2.1 Data Sources

This meta‐analysis was registered with the International Prospective Register of Systematic Reviews (PROSPERO) on October 12, 2024, and its registration was approved on October 23, 2024 (CRD42024600740). The meta‐analysis followed the Preferred Reporting Items for Systematic Reviews and Meta‐Analyses (PRISMA Statement) (Page et al. 2021). The Embase, LILACS, PsycInfo, PubMed, Scopus, and Web of Science databases were searched for relevant articles (search last updated on April 29, 2025), using the following combination of search terms in all databases: Empathy AND Nursing AND (Burnout OR “Burnout Syndrome” OR “Psychological Burnout”).

### 2.2 Eligibility Criteria

We included original, quantitative, cross‐sectional, and longitudinal articles that investigated relationships between empathy and/or the subdomains of affective or cognitive empathy and burnout syndrome or emotional exhaustion (i.e., the main indicator of burnout syndrome) among nurses in any field. No restrictions were imposed regarding language or date of publication. Studies that did not perform correlation analysis between variables (i.e., empathy or its subdomains and burnout syndrome), that used heterogeneous samples composed of different healthcare providers, and studies with repeated samples were excluded.

### 2.3 Data Extraction

Rayyan software (https://www.rayyan.ai/) was used, which is an online reference manager developed to assist in the steps of systematic reviews (Ouzzani et al. 2016). After extracting data to the reference manager, two reviewers (MAM‐B and AOR) identified and excluded duplicate articles, followed by the preselection of articles based on an analysis of titles and abstracts. All pre‐selected articles were submitted to full‐text analysis. Articles that generated doubts between the reviewers (MAM‐B and AOR) either individually or collectively were brought to a third reviewer (BML), who made the final decision regarding inclusion or exclusion according to the established eligibility criteria.

**FIGURE 1:**
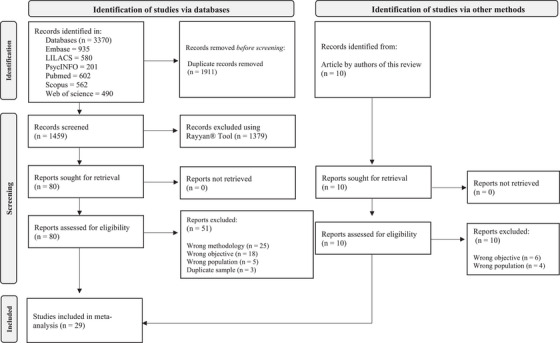
Flow diagram.

To minimize the possible non‐inclusion of relevant articles (Harari et al. 2020) and following the recommendations of the PRISMA Statement (Page et al. 2021), the reviewers manually searched the reference lists of the articles selected for the meta‐analysis. All the steps described above were performed independently to avoid selection bias (McDonagh and Peterson 2014). Strategies for gaining access to the full text of all selected articles were also pre‐established, such as email requests to the corresponding author, requests to authors via ResearchGate, and voluntary collaboration from researchers of other countries. Figure 1 displays all steps of the article selection process in detail.

### 2.4 Data Synthesis

The following information was extracted from the articles that met the eligibility criteria by two reviewers (MAM‐B and AOR): (i) first author; (ii) year of publication; (iii) country in which the study was conducted; (iv) study design; (v) sex of the participants; (vi) mean age of the participants; (vii) number of participants; (viii) study location; (ix) specialties of the nurses (e.g., mental health, oncology, etc.); (x) length of work experience; (xi) measures of empathy; (xii) measures of burnout syndrome; (xiii) relationships between global empathy, its domains (i.e., affective and cognitive) and subdomains (i.e., empathic concern, personal distress, perspective taking, and fantasy) and burnout syndrome.

### 2.5 Interrater Reliability

Given the subjectivity of each reviewer in the different steps of the review process, which may imply a lack of agreement, a statistical assessment was performed using Cohen's Kappa coefficient, which is widely used in systematic reviews (McHugh 2012). The coefficient is interpreted as follows: 0 = no agreement; 0.01–0.20 = slight, 0.21–0.40 = fair, 0.41–0.60 = moderate, 0.61–0.80 = substantial, and ≥ 0.81 = perfect agreement (Cohen 1960; McHugh 2012). Moderate inter‐reviewer reliability was found in the selection step based on the analysis of titles and abstracts (κ = 0.60; *p* < 0.001; 96.2% agreement). Substantial reliability was found in the step involving full‐text analysis (κ = 0.62; *p* < 0.001; 82.8% agreement). These statistical analyses were performed using SPSS software version 30.0.

### 2.6 Critical Appraisal

The checklist of the Joanna Briggs Institute (JBI) Critical Appraisal Tools was used to assess the methodological quality of the selected studies. The JBI instrument was chosen because it is among the three most recommended for the assessment of risk of bias (Ma et al. 2020). The checklist has eight questions, each with answers on a Likert scale (i.e., Yes, No, Unclear, and Not Applicable) (Moola et al. 2017). Risk of bias is classified based on the percentage of “Yes” answers, with lower percentages denoting a greater risk of bias: ≥ 70% = low; 50 to 69% = medium, and ≤ 49% high risk of bias. The classification identified in other systematic reviews was adopted (see Maximiano‐Barreto et al. 2024a; Lima et al. 2024). The appraisal was performed by two authors (BML and MM) working independently, and the analysis of disagreements was carried out among three authors (MAM‐B, BML, and MM).

### 2.7 Meta‐Analysis

The Jamovi 2.3.28 software program was used to perform the meta‐analysis, with the determination of the effect size considering correlation coefficients and sample size. Five meta‐analyses were conducted separately, considering (1) global empathy, (2) empathic concern, (3) personal distress, (4) perspective taking, and (5) fantasy. All of these aspects were analyzed in relation to burnout syndrome and/or emotional exhaustion, which is the main subdomain of burnout syndrome and the most widely investigated (see Aronsson et al. 2017; O'Connor et al. 2018). The raw data of the correlation values are displayed in Supplementary Table S1. The effect size was calculated in each meta‐analysis considering a 95% confidence interval.

A random‐effects meta‐analysis was conducted, considering the inherent heterogeneity among the studies. The DerSimonian–Laird estimator was used (DerSimonian and and Laird 1986; DerSimonian and Kacker 2007). Heterogeneity among the studies was assessed using the Q test (Cochran 1954; Higgins et al. 2002) and the I^2^ statistical test (Higgins et al. 2002), with heterogeneity classified as high, moderate, or low (i.e., I^2^ of 60%–90%, 40%–59%, and 0%–39%, respectively) (Borenstein et al. 2009), potential sources of heterogeneity (e.g., cultural context, measurement tools). The risk of publication bias was assessed using funnel plots. Egger's and Begg's tests (Begg and Mazumdar 1994; Egger et al. 1997) were used to quantitatively assess the extent of asymmetry. Due to the limited number of studies included in the meta‐analyses of empathy subdomains, these findings should be interpreted with caution. The effect size of the correlation was classified as absent/weak, moderate, strong, or very strong (0.0–0.25, 0.26–0.50, 0.51–0.70, and ≥ 0.71, respectively) (Streiner and Norman 2003).

## 3 Results

A total of 3,370 articles were identified in the databases, 29 of which met the eligibility criteria. Although a manual search of the reference lists of these 29 articles identified 10 possible articles for inclusion, none of these articles met the previously defined criteria. The entire article inclusion and exclusion process is displayed in Figure 1.

### 3.1 Characteristics of Studies

The first study identified in this meta‐analysis was published in the early 1990s (Åström et al. 1990). However, the largest concentration of studies is found in the last 10 years. The studies were developed on four of the five continents (i.e., America, Asia, Europe, and Oceania); however, more than half of the studies (*n* = 16) were conducted in Asian countries. Regarding the design, 28 were cross‐sectional studies and only one was a longitudinal study (Altmann and Roth 2021). A total of 11,971 nurses were included in this meta‐analysis. The smallest sample had 64 nurses (Caro et al. 2017), and the largest had 1,236 (Pérez‐Fuentes et al. 2019). The average proportion of female nurses was 87.0% (*n* = 10,412). Mean age ranged from 22.45 ± 1.02 years (Cao et al. 2021) to 46.10 ± 12.73 years (Serrada‐Tejeda et al. 2025). However, the mean age in most studies was between 30 and 39 years.

Fifteen of the 29 articles were conducted with nursing staff from different fields. In 13 studies, the samples were composed of nurses from a single specialty, namely: geriatrics (*n* = 3) (Åström et al. 1990; Narme 2018; Serrada‐Tejeda et al. 2025), oncology (*n* = 2) (Shi et al. 2022; Taleghani et al. 2017), mental health (*n* = 2) (Román‐Sánchez et al. 2022; Wilczek‐Ruzyczka 2020), emergency care (*n* = 2) (Salvarani et al. 2019; Yu et al. 2021), palliative care (*n* = 2) (Caro et al. 2017; Kayikci et al. 2025), public health (*n* = 1) (Kitano et al. 2023), and surgery (*n* = 1) (Załuski et al. 2020). Most studies (86.2%) involved nurses who worked in hospital settings. Length of experience among the nurses was up to ten years in five studies and between 10 and 20 years in 11 studies. The characteristics of the studies are displayed in detail in Table 1.

**TABLE 1:** Sample and methodological characteristics of the studies included in the meta‐analysis.

Reference	Country	Design	N	Sex, female (%)	Mean age (± SD)	Specialty of nurses	Setting	Length of work experience	Empathy instrument	Burnout instrument
Altmann (2021)	Germany	L	172	(81.50)	38.8 (±11.3)	Different specialties	Hospital	≥10 to ≤20 years	TEQ	CBI
Åström et al. (1990)	Sweden	CS	358	(88.80)	32.0	Geriatrics	Psychogeriatric clinic; Somatic long‐term care; Nursing home	≥10 to ≤20 years	ECRS	BS
Cao et al. (2021)	China	CS	393	(81.90)	22.45 (±1.02)	Different specialties	Hospital	−	JSE	ProQOL
Caro et al. (2017)	Chile	CS	64	(83.00)	40 (±10)	Palliative care; Clinical nurse	Home care	−	JSE‐HP	MBI‐HSS
Cheng et al. (2020)	China	CS	363	(97.80)	29.33 (±6.16)	Different specialties	Hospital	−	SECCN	MBI‐GS
Dor et al. (2019)	Israel	CS	457	(78.00)	37.30 (±6.16)	Different specialties	Hospital	≥10 to ≤20 years	TEQ	MBI
Duarte et al. (2016)	Portugal	CS	280	(81.10)	37.66 (±9.34)	Different specialties	Hospital	≥10 to ≤20 years	IRI	ProQOL‐5
Fitzgerald‐Yau et al. (2018)	Ireland	CS	442	(93.20)	41.1 (±9.8)	Different specialties	−	≥10 to ≤20 years	BLRI	MBI
Gountas and Gountas (2016)	Australia	CS	159	(78,00)	−	Different specialties	Hospital	−	IRI	EE (Wharton 1993)
Hui et al. (2020)	China	CS	733	(100,00)	27.0 (±4.1)	Different specialties	Hospital	≥10 to ≤20 years	IRI	ProQOL‐5
Kayikci et al. (2025)	Turkey	CS	141	(80.1)	30.53 (±6.57)	Palliative care	Hospital	—	ETS	ProQOL
Kitano et al. (2023)	Japan	CS	1.006	(97.20)	−	Public health	City hall	<10 years	IRI	MBI
Mersin et al. (2024)	Turkey	CS	653	(88.7)	34.86 (±9.27)	Different specialties	Hospital	—	BES	BMS
Narme (2018)	France	CS	124	(92.70)	38.4 (±11.30)	Geriatrics	LSIOP	<10 years	IRI	MBI
Pérez‐Fuentes et al. (2019)	Spain	CS	1236	(84.50)	31.50 (±6.18)	Different specialties	Hospital	>20 years	EHC−PS	CBB
Raižiene et al. (2007)	Lithuania	CS	158	(100)	41.60 (±8.03)	Different specialties	Hospital	−	IRI	MBI
Ren et al. (2020)	China	CS	786	(97.96)	31.89 (±13.32)	Different specialties	Hospital	≥10 to ≤20 years	IRI	MBI‐GS
Román‐Sánche et al. (2022)	Spain	CS	750	(62.50)	−	Mental health	Hospital	>20 years	JSE	MBI
Şahin et al. (2018)	Turkey	CS	334	(88.00)	30.22 (±6.17)	Different specialties	Hospital	>20 years	JSE	MBI
Salvarani et al. (2019)	Italy	CS	97	(61.86)	38.00 (±9.48)	Emergency care	Hospital	<10 years	IRI	MBI
Serrada‐Tejeda et al. (2025)	Spain	CS	104	(87.50)	46.10 (±12.73)	Geriatrics	Nursing center	≥10 to ≤20 years	IRI	ProQOL‐CSF‐IV
Shi et al. (2022)	China	CS	794	(98.20)	31.57 (±7.12)	Oncology	Hospital	>20 years	IRI	ProQOL
Taleghani et al. (2017)	Iran	CS	67	(88.06)	30.39 (±8.35)	Oncology	Hospital	<10 years	JSE	MBI
Topçu et al. (2023)	Turkey	CS	434	(78.80)	−	Different specialties	Hospital	<10 years	TEQ	SBI
Wilczek‐Ruzyczka (2020)	China	CS	186	(93.00)	28.33 (±5.61)	Emergency care	Hospital	<10 years	IRI	MBI
Ye et al. (2024)	China	CS	221	(91.40)	21.87(±1.50)	Different specialties	Hospital	—	JSE	ProQOL
Yıldırım et al. (2024)	Turkey	CS	1189	(86.60)	32.75 (± 8.53)	Different specialties	Hospital	≥10 to ≤20 years	PSF	ProQOL
Yu et al. (2021)	China	CS	186	(93.00)	28.33 (±5.61)	Emergency care	Hospital	<10 years	JSE	ProQOL
Załuski et al. (2020)	Poland	CS	84	−	−	Surgical care	Hospital	−	EQ−short	LBQ

Abbreviations: −, Not reported; BES, Basic Empathy Scale; BLRI, Barrett−Lennard Relationship Inventory; BMS, Burnout Measure Short Scale; BS, Burnout Scale; CBB, Brief Burnout Questionnaire; CBI, Copenhagen Burnout Inventory; CS, cross‐sectional; EASHN, Empathy Ability Scale for Hospice Nurses; ECRS, Empathy Construct Rating Scale; EHC−PS, Communication Skills Scale for Healthcare Professionals; EQ−short, Empathy Quotient—short; IRI, Interpersonal Reactivity Index; IS, Irritation Scale; ISEI, Interpersonal and Social Empathy Index; JSE, Jefferson Scale of Empathy; JSE−HP, Jefferson Scale of Empathy—Health Professionals; L, longitudinal; LBQ, Link Burnout Questionnaire; LSIOP, long‐stay institution for older people; MBI, Maslach Burnout Inventory; MBI−GS, Maslach Burnout Inventory—General Services; MBI−HSS, Maslach Burnout Inventory—Human Services Survey; ProQOL, Professional Quality of Life Scale; ProQOL−5, The Professional Quality of Life Scale, version 5; PSF, Psychosocial Skills Form; SBI, Spanish Burnout Inventory; SECCN, Scale of Empathy Competencies of Clinical Nurses; TEQ, Toronto Empathy Questionnaire; TES, Toronto Empathy Scale; TRIG, Texas Revised Inventory of Grief.

### 3.2 Instruments Used for Assessment of Burnout Syndrome

Indicators of burnout syndrome were investigated using 10 different instruments. The Maslach Burnout Inventory (different versions), Burnout Measure Short Scale, and Link Burnout Questionnaire were used to assess the three main indicators of burnout syndrome (e.g., emotional exhaustion, depersonalization, and personal fulfillment). The Brief Burnout Questionnaire is used to assess the occurrence of emotional exhaustion in nurses. The Burnout Scale is used to assess physical, mental, or emotional burnout syndrome, and the Copenhagen Burnout Inventory is used to assess physical and psychological symptoms. It should be noted that “personal burnout” measured by the Copenhagen Burnout Inventory refers to emotional exhaustion. The Professional Quality of Life Scale (ProQOL) is a nonspecific measure for assessing burnout syndrome but has a subdomain for the global assessment of burnout syndrome. However, the inventory developed by Maslach was the most widely used (13 studies). The ProQOL was used in eight of the articles that compose the present review, although it is not a specific measure for the assessment of burnout syndrome.

### 3.3 Instruments Used for Assessment of Empathy

Fourteen different measures were used to assess empathy levels in nurses. Besides assessing global empathy and its affective and cognitive domains, the measures are also used to assess the subdomains of this construct. Three are used to investigate empathic concern (i.e., Scale of Empathy Competencies of Clinical Nurses, Interpersonal Reactivity Index, and Jefferson Scale of Empathy) and two are used to investigate personal distress (i.e., Toronto Empathy Questionnaire and Interpersonal Reactivity Index). Fantasy is measured using a single scale (i.e., Interpersonal Reactivity Index) and perspective taking is measured on two (i.e., Jefferson Scale of Empathy and Interpersonal Reactivity Index). One study discriminated empathic concern as an “emotional experience” measured by the Scale of Empathy Competencies of Clinical Nurses (Cheng et al. 2020). The Interpersonal Reactivity Index (IRI) and Jefferson Scale of Empathy (JSE) are the most commonly used.

### 3.4 Quality of Studies

Twenty‐five of the 29 articles had a low risk of bias, with the score ranging from 75% to 100%. Ten of these articles had no risk of bias (100% fulfillment of items). Among the weak points identified, 17 studies did not report strategies for dealing with confounding variables, such as age, length of work experience, gender, and marital status. Details of the risk‐of‐bias appraisal of the 29 articles are displayed in Supplementary Table S2.

### 3.5 Correlation Between Empathy and Burnout Syndrome

**FIGURE 2:**
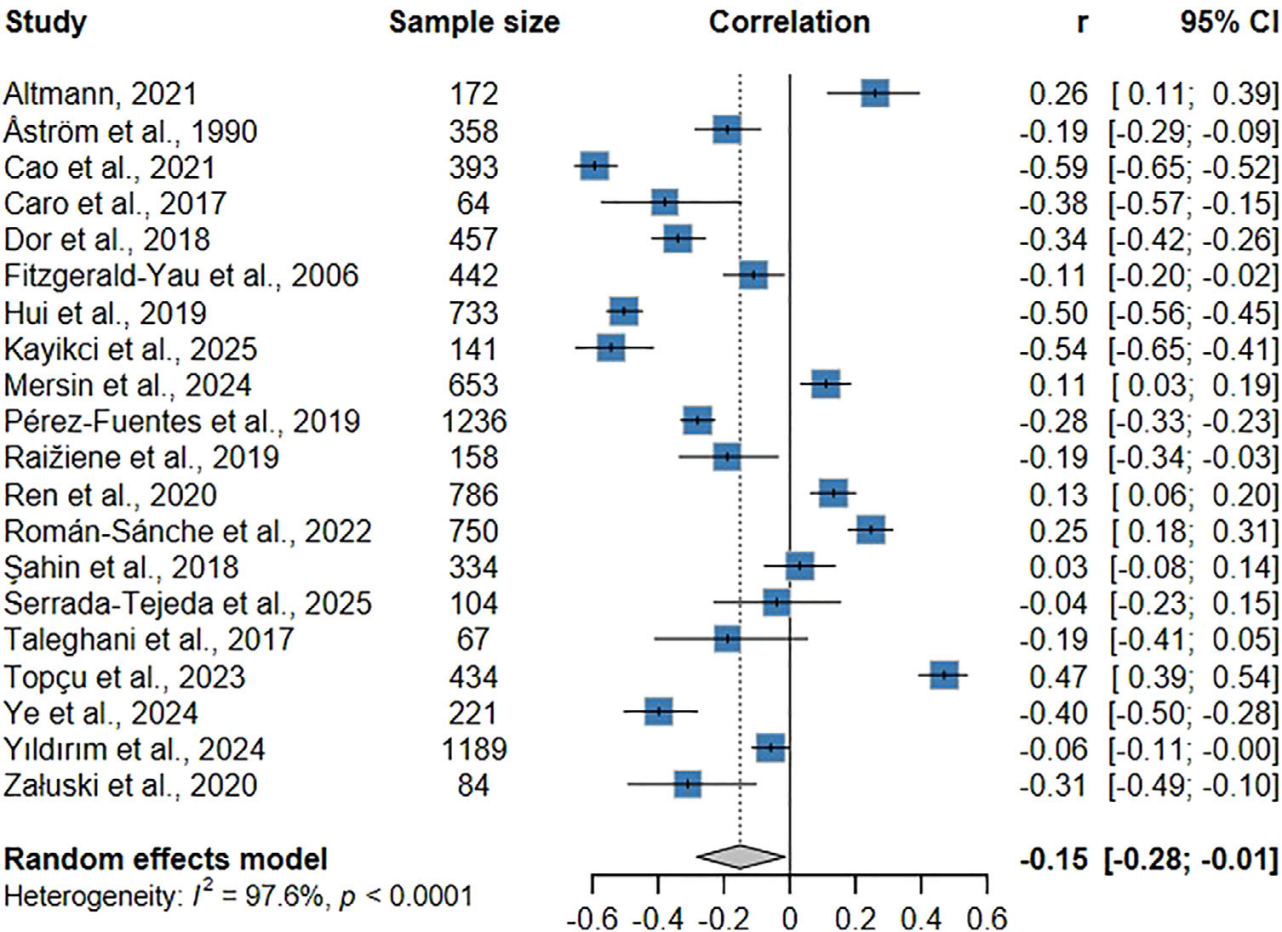
Forest plot of the meta‐analysis examining the association between empathy and burnout syndrome.

Figure 2 displays the results of the meta‐analysis of global empathy and burnout syndrome or emotional exhaustion (the main indicator of burnout syndrome), which was investigated in 20 articles that described raw correlation values between the variables. The meta‐analysis identified a weak, negative, statistically significant correlation between these constructs (*r* = −0.15; 95% CI: −0.29 to −0.01; *p* = 0.03). This result suggests that higher levels of empathy are correlated with lower indicators of burnout syndrome. The results also indicate high heterogeneity among the studies (I^2^ = 97.57%; Q test: *p* < 0.001). Supplementary Figure S1a displays the funnel plot of this meta‐analysis qualitatively, indicating no publication bias. Risk of bias assessed quantitatively was not identified using Egger's and Begg's tests (*p*‐value > 0.05).

### 3.6 Correlation Between Subdomains of Affective Empathy and Burnout Syndrome

#### 3.6.1 Empathetic Concern

**FIGURE 3:**
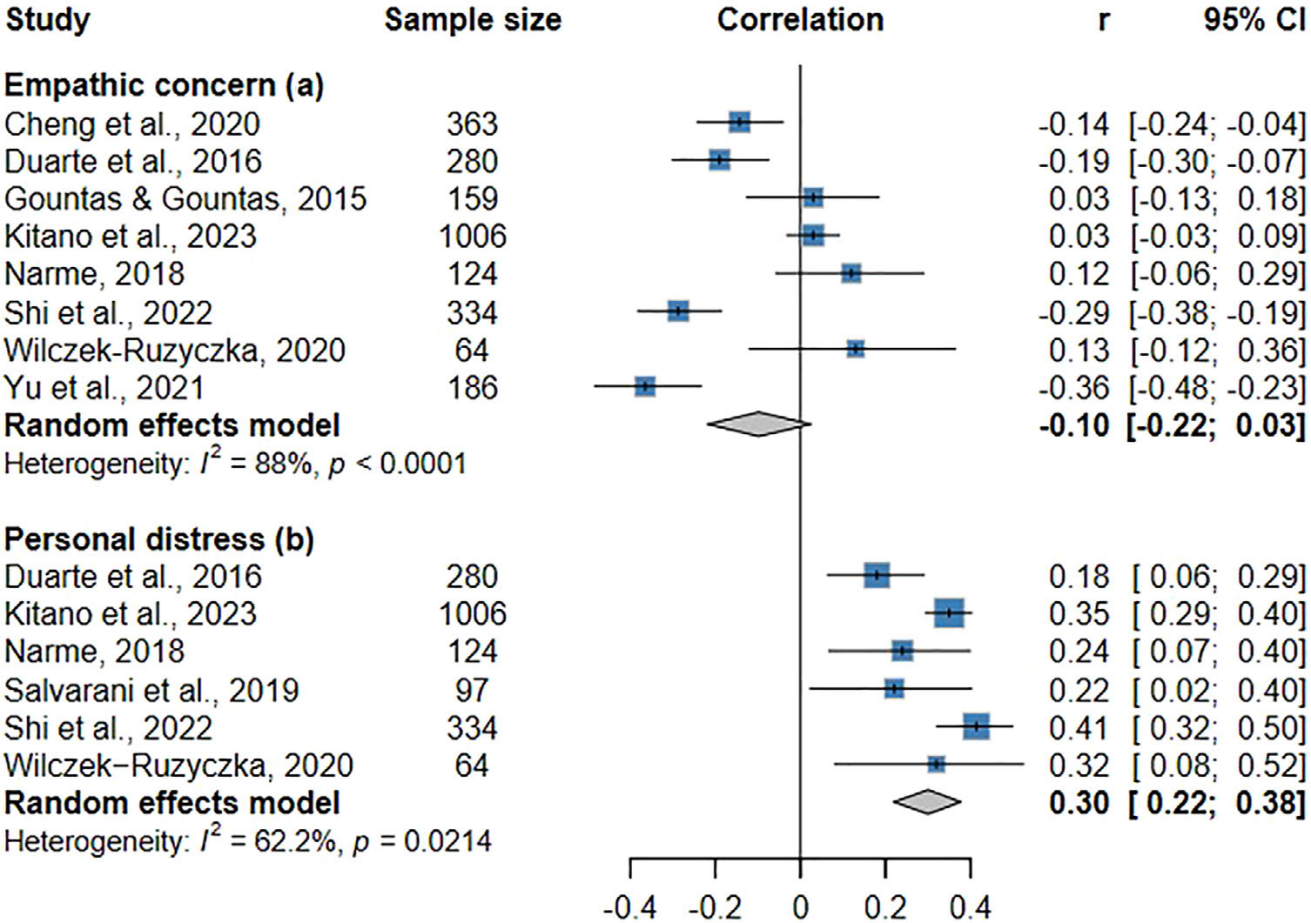
Forest plot of the meta‐analysis examining the association between burnout syndrome and affective empathy: empathic concern (a) and personal distress (b).

Eight studies investigated the relationship between empathic concern and burnout syndrome or its main indicator (i.e., emotional exhaustion) (Figure 3a). The meta‐analysis revealed no statistically significant effects (*r* = −0.10; 95% CI: −0.22 to 0.03; *p* = 0.27). High heterogeneity was found among the studies (I^2^ = 88.0%; *p* < 0.01). Supplementary Figure S1b shows good dispersion among data points and suggests an absence of publication bias. Egger's (*p* = 0.905) and Begg's (*p* = 0.331) tests quantitatively demonstrated the absence of risk of bias.

#### 3.6.2 Personal Distress

Six articles described raw correlation values between personal distress and burnout syndrome or its main subdomain (emotional exhaustion) (Figure 3b). A statistically significant, weak, tending to moderate effect was identified (*r* = 0.30; 95% CI: 0.22–0.38; *p* = 0.01). This finding suggests that higher levels of personal distress lead to higher indicators of burnout among nurses. Heterogeneity (I^2^ = 62.2%; *p* = 0.02) was high across the studies. The funnel plot in Supplementary Table S1c qualitatively demonstrates a low risk of bias. Egger's and Begg's tests quantitatively demonstrated no risk of bias (*p* > 0.05).

### 3.7 Correlation Between Subdomains of Cognitive Empathy and Burnout Syndrome

#### 3.7.1 Perspective Taking

**FIGURE 4:**
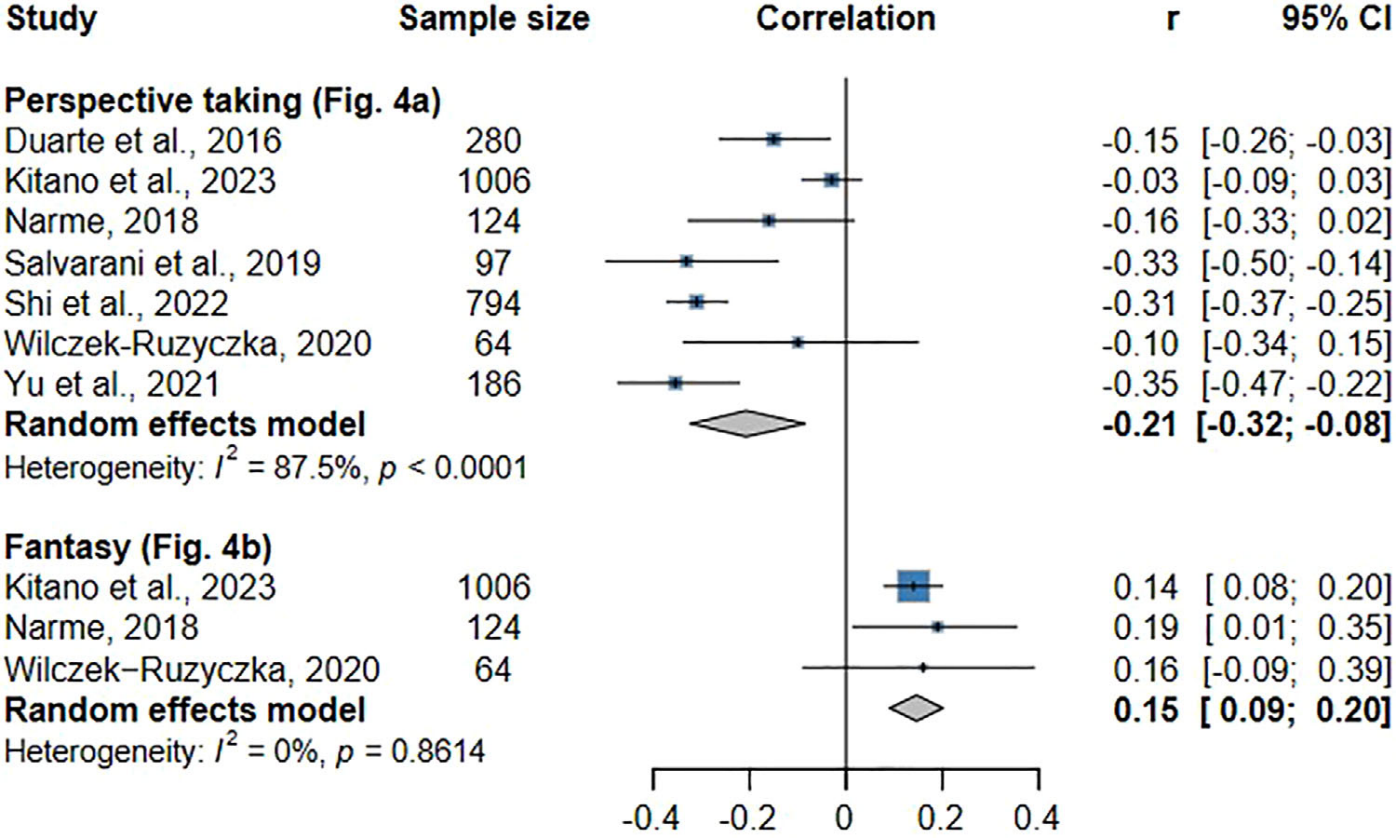
Forest plot of the meta‐analysis examining the association between burnout syndrome and cognitive empathy: perspective taking (a) and fantasy (b).

The correlation between perspective taking and burnout syndrome or emotional exhaustion was reported in seven articles included in this meta‐analysis (Figure 4a). The results show a negative, statistically significant, weak correlation between perspective taking and burnout syndrome (*r* = −0.21; CI: −0.32 to −0.08; *p* = 0.01). This result indicates that higher levels of perspective taking lead to lower indicators of burnout syndrome. Heterogeneity among the studies was high (I^2^ = 87.5%; *p* < 0.01). The funnel plot in Supplementary Figure S1d revealed no quantitative risk of publication bias (*p* > 0.05 for both Egger's and Begg's tests).

#### 3.7.2 Fantasy

A weak but positive statistically significant correlation was found between the variables (*r* = 0.15; CI: 0.09–0.20; *p* = 0.01), showing that higher levels of fantasy are correlated with increased indicators of burnout syndrome among nurses (Figure 4b). Low heterogeneity was found among the studies (I^2^ = 0.0%; *p* > 0.05), indicating statistical consistency. The qualitative risk assessment indicated publication bias, which may be explained by the absence of studies with a negative effect. In the quantitative analysis, however, Egger's and Begg's tests indicated no risk of bias, as the *p*‐values were not statistically significant (*p* > 0.05).

## 4 Discussion

The meta‐analyses performed in this review sought to test two previously established hypotheses (i.e., higher levels of global empathy as well as the subdomains of affective empathy correlate positively with burnout syndrome, whereas the subdomains of cognitive empathy correlate negatively). The findings suggest that the hypotheses were partially confirmed, as global empathy was negatively associated with burnout syndrome. Regarding the subdomains of affective empathy, no significant correlation was found with empathic concern, whereas personal distress was positively correlated, indicating that higher levels of such experiences are associated with burnout syndrome. Concerning the subdomains of cognitive empathy, a significant negative correlation was found with perspective taking (higher levels of perspective taking are negatively correlated with indicators of burnout syndrome), whereas fantasy was positively correlated.

The negative correlation between global empathy and burnout syndrome in nurses in the present study is in disagreement with findings identified in previous systematic reviews conducted with nurses (Hunt et al. 2017), paid and unpaid caregivers of older people (Maximiano‐Barreto et al. 2024a), and meta‐analyses involving medical students (Cairns et al. 2024), healthcare professionals (Delgado et al. 2023; Martingano et al. 2025), and non‐healthcare professionals (Martingano et al. 2025). This divergence underscores the importance of continuing to investigate the relationship between these two variables. However, low pay and precarious working conditions, among other factors (Duquette et al. 1994; Dall'Ora et al. 2020), may explain these findings, which, in turn, contribute to higher levels of burnout syndrome (see Kamanzi and Nkosi 2011) in this population and exert an impact on lower levels of empathy (Yu et al. 2022).

Another factor that could contribute to the negative association between global empathy and burnout syndrome is the different fields in which nurses practice. The review by Hunt et al. (2017) included only five articles and excluded studies involving nurses from specific fields (e.g., mental health). The present meta‐analysis, however, sought to address this by including all fields of nursing practice and a considerable number of studies (i.e., 29). However, a study involving 112 nurses working in different sectors (i.e., psychiatry, emergency care, and intensive care) found no difference in empathy levels (Ghaedi et al. 2020). It should be noted that the nurses had more than 10 years of experience in most of the studies selected for the present meta‐analysis. The experience of healthcare providers (e.g., nurses) can contribute to an increase in empathy levels (Maximiano‐Barreto et al. 2020). Conversely, less experienced nurses are more likely to develop burnout syndrome (Lee et al. 2024), as identified in a study carried out with 5,956 nurses (Kanai‐Pak et al. 2008).

The nurses in this meta‐analysis worked in different sectors (e.g., emergency care and mental health). Studies have shown that the subdomains of affective empathy are activated differently depending on whether the target subject (e.g., patient) has emotional and/or physical issues (Decety and Ickes 2009; Wondra and Ellsworth 2015). These findings underscore the importance of assessing these subdomains in nurses, who often deal with patients who have both emotional and physical needs (Bay et al. 2025). Moreover, meta‐analyses conducted with unpaid caregivers of older people (Huo et al. 2025) and healthcare professionals (Delgado et al. 2023) found distinct results between the subdomains of both affective and cognitive empathy.

Empathic concern is one of the subdomains of affective empathy, which involves caring and concern for the well‐being of the target subject (e.g., patient) (Davis 1980). This is a fundamental skill for nurses, whose work typically focuses on providing care for sick people to minimize their suffering (Younas and Inayat 2024) and promote their well‐being (Wilson and Leese 2013). A previous meta‐analysis conducted with caregivers of older people found similar results, that is, no statistically significant association between empathic concern and psychological concerns (e.g., burnout syndrome, anxiety, or depression) (Huo et al. 2025). However, it is important to understand the relationship between this construct and burnout syndrome as well as other psychological concerns. Although some studies suggest interventions aimed at reducing levels of affective empathy (e.g., Hua et al. 2021), it is actually necessary to balance the two main domains of empathy, as suggested in a recent intervention study (see Maximiano‐Barreto et al. 2024b). Furthermore, empathic concern is a subdomain of affective empathy, which is important in the caregiver‐care recipient relationship and better predicts prosocial behavior compared with other subdomains (Kamas and Preston 2021).

The relationship between affective empathy and psychological concerns (e.g., burnout syndrome) identified in previous studies (e.g., Cairns et al. 2024; Hunt et al. 2017; Huo et al. 2025; Maximiano‐Barreto et al. 2024a) may be explained by another subdomain of affective empathy (i.e., personal distress). These divergent results demonstrate the importance of deepening the understanding of these subdomains of affective empathy. Empathic concern differs from personal distress, as the latter is related to the ability of the subject (e.g., nurses) to experience the emotions and feelings of others (e.g., patients). Moreover, this subdomain enables the empathizer to alleviate their own pain, but it is first necessary to alleviate the other person's pain (Davis 1980, 1983). Nurses are in daily contact with patients who have physical and/or emotional issues. As described above, personal distress responds better than empathic concern in situations involving physical and emotional pain (Decety and Ickes 2009; Wondra and Ellsworth 2015), which may explain the direct relationship between personal distress and burnout syndrome.

Empathy involves both behavioral and physiological processes (Qaiser et al. 2023), and the relationship between these variables (i.e., personal distress and burnout syndrome) may also be explained from a physiological standpoint. Mirror neurons are associated with affective empathy (Haeusser 2012). These neurons enable the empathizer to perform subjective resonance with the target individual, in addition to activating the same regions of the brain in the empathizer when observing the target individual (e.g., when the target individual cries, the empathizer also cries) (Carr et al. 2003). Personal distress involves the experience of positive and negative emotions and feelings (Davis 1980). Paz et al. (2022) found that mirror neurons are related to contagious depression (i.e., contact with a patient with depression can lead to others—e.g., nurses—having depression), which we hypothesize can also occur in burnout syndrome, leading nurses to emotional exhaustion.

Along the same line, the amygdala exhibits greater activation when the empathizing subject imitates the target subject (Carr et al. 2003). This region of the brain is one of the specific areas related to affective empathy (Derntl et al. 2010), more specifically to personal distress, as distinct from empathic concern (see: Ho et al. 2014). The amygdala is also activated in individuals experiencing burnout syndrome (Bayes et al. 2021; Khammissa et al. 2022). This is another aspect that may explain the association between personal distress and burnout syndrome among nurses identified in one of the meta‐analyses conducted.

Just as the subdomains that comprise affective empathy were associated differently with burnout syndrome, the same occurred with the subdomains of cognitive empathy. However, previous studies have shown that cognitive empathy is negatively associated with burnout syndrome (Cairns et al. 2024; Huo et al. 2025; Maximiano‐Barreto et al. 2024a). With regard to perspective taking, the findings of this meta‐analysis are similar to those identified in the meta‐analysis conducted by Huo et al. (2025) and Delgado et al. (2023). However, it is important to bear in mind that the authors analyzed unpaid caregivers of older people. This subdomain of cognitive empathy enables one to distance oneself from the target subject (i.e., not experience the same feelings and emotions) (Davis 1980, 1983). Nurses who can distance themselves from their patients’ feelings and emotions can help avoid compassion fatigue (Slatten et al. 2011) and thus protect themselves from burnout, as these variables are directly associated (see Vancampfort and Mugisha 2022). A study involving physicians found that those with higher levels of perspective taking had greater clinical empathy abilities and lower levels of burnout (Thirioux et al. 2016). This is expected, as burnout can negatively impact the quality of care and the healthcare provider‐patient relationship (Dall'Ora et al. 2020).

Considering the negative association between perspective taking and burnout syndrome, Ho et al. (2014) found that perspective taking was associated with lower cortisol levels, unlike personal distress, which may explain this distinction in the results identified in the present investigation. Other studies have also sought to assess the association between empathy and cortisol levels (e.g., Azulay et al. 2022; Tang et al. 2024). Recent studies conducted with healthcare providers also found that higher cortisol levels were associated with burnout syndrome (Fernández‐Sánchez et al. 2018; Marcil et al. 2022). These aspects demonstrate the importance of thinking about not only non‐pharmacological interventions (see Maximiano‐Barreto et al. 2024c) but also pharmacological interventions (e.g., cannabidiol) (Sainz‐Cort et al. 2024) to improve aspects related to empathy and psychological concerns (e.g., burnout syndrome) (Crippa et al. 2021).

Fantasy, which is a subdomain of cognitive empathy, has been investigated little. The lack of the assessment of this subdomain of cognitive empathy may be explained by a possible divergence in the understanding of this domain as belonging to empathy. For instance, the exclusion of the fantasy domain was suggested in a study on the factorial structure of the IRI in a Brazilian sample, given that the items and domain did not fit the proposed model (Koller and Camino 2001). In a 1992 oral presentation, Eisenberg suggested the exclusion of this subdomain of empathy, as cultural aspects influenced the development of the IRI, which is the main measure of empathy and the only one that addresses the fantasy subdomain. In a meta‐analysis, this subdomain was positively correlated with burnout syndrome among nurses. A study conducted with 10,303 individuals 10 to 88 years of age using the IRI suggested that fantasy is more appropriate as a construct belonging to the affective domain of empathy (Paulus 2024). Moreover, the author found that this subdomain correlated directly with the subdomains of affective empathy. It was also found that women score higher on this construct compared with men (Gilet et al. 2013; Lucas‐Molina et al. 2017), which may explain the findings, given that most of the selected studies consisted of female nurses. Nurses may imagine themselves in situations similar to those of their patients, which is an aspect of this subdomain of empathy (see Davis 1980, 1983), increasing levels of the fantasy subdomain and concomitantly leading to burnout syndrome. These findings demonstrate the importance of training focused on cognitive empathy (see Maximiano‐Barreto et al. 2024c) and, more specifically, on perspective taking (see Au et al. 2020).

Some weaknesses observed in the literature analyzed should be considered. Different measures were used to assess the constructs, primarily self‐reports, which may imply socially desirable responses and may have favored the high heterogeneity in the meta‐analyses. Although the scale developed by Davis (1983) (i.e., IRI) is widely used, there is no gold standard for measuring empathy (Lima and Osório 2021), especially among nurses (Yu and Kirk 2009). Moreover, the measures lack convergent validity (Lima and Osório 2021). The lack of analyses on the subdomains of affective and cognitive empathy demonstrates that, in addition to assessing affective and cognitive empathy, it is also important to understand how these subdomains interact, not only with burnout syndrome, but with other psychological concerns (e.g., anxiety and depression). The inclusion of different nursing specialties also constitutes a limitation, as this meta‐analysis was not yet able to cover all fields of nursing. However, unlike the previous review by Hunt et al. (2017), a considerable number of specialties were included. Another noteworthy fact is the inclusion of mainly cross‐sectional studies, which demonstrates the need for longitudinal studies for a better understanding of the effects of the subdomains that comprise empathy and burnout syndrome in nurses over time.

With regard to the limitations of this review, the use of a search strategy exclusively in English may have led to the exclusion of studies in the literature due to a lack of titles and abstracts in this language (i.e., English). However, articles published in other languages (e.g., Spanish and French) were selected in this review, which reduces the occurrence of publication bias. The high heterogeneity observed in the meta‐analyses also constitutes a limitation that should be considered. This aspect underscores the need for caution in interpreting the findings because, in addition to the factor previously mentioned (i.e., use of different measurement instruments), cultural context and varying levels of nurses’ professional experience should be taken into account.

Despite the limitations, this meta‐analysis offers significant contributions to the field of nursing and is one of the first meta‐analyses to comprehensively examine the association between global empathy and its subdomains (i.e., empathic concern, personal distress, perspective taking, and fantasy) and burnout syndrome in nurses. The results suggest the need to expand investigations into these subdomains of empathy and suggest the importance of interventions focusing on perspective taking, which is the main subdomain of cognitive empathy and serves as a protection factor against burnout syndrome. Affective empathy and its subdomains are important constructs in the nurse–patient relationship and therefore cannot be dismissed. Lastly, the findings indicate the importance that healthcare managers should attach to this ability and how it can impact the mental health of nurses.

## 5 Implications for Nursing and Health Policy

Understanding the distinct interactions that the subdomains of affective and cognitive empathy have with burnout syndrome highlights the importance of attention from both nurses and health managers to these professionals, given that this ability is important in the nurse–patient relationship. The implementation of interventions focusing on this ability, especially in perspective taking, a subdomain of cognitive empathy, may constitute another strategy to be implemented to minimize the mental health impacts on nurses.

## Author Contributions

MAM‐B and FLO designed the meta‐analysis. MAM‐B and AOR conducted the literature search, selected the studies, and extracted data from the primary studies. MM and BML performed the analysis of the methodological quality of the selected studies. All other authors assisted in solving issues. FLO and MAM‐B conducted the analysis. MAM‐B wrote the first draft of the manuscript. MAM‐B, AOR, MM, MHNC, MBL, and FLO contributed to and approved the final manuscript.

## Conflicts of Interest

The authors declare no conflicts of interest.

## Funding

This study was financed in part by the Brazilian fostering agency *Coordenação de Aperfeiçoamento de Pessoal de Nível Superior* (CAPES [Coordination for the Advancement of Higher Education Personnel])—Finance Code 001). Maximiano‐Barreto MA is a recipient of a scholarship from *Conselho Nacional de Desenvolvimento Científico e Tecnológico* (CNPq [National Council for Scientific and Technological Development])—(Process No: 150062/2024‐9; 402148/2024‐0) and *Fundação de Amparo à Pesquisa do Estado de São Paulo* (FAPESP [State of São Paulo Research Assistance Foundation (process: 2024/02908‐4)]). Osório FL is a CNPq–Productivity Research Fellow—(Process No: 302225/2022–6). Luchese BM is a CNPq–Productivity Research Fellow—(Process No: 308019/2023‐7). Chagas MH is the recipient of a CNPq Research Productivity Fellowship (Process no.: 309870/2025‐9). The participation of Matias M in this work was supported by national funding from the Portuguese Foundation for Science and Technology (https://doi.org/10.54499/UID/00050/2025).

## Ethics Statement

As this is a meta‐analysis based on previously published studies, it is exempt from approval by an Ethics Committee.

## Supporting information




**Figure S1**: Funnel plot assessing publication bias in the meta‐analyses of overall empathy (a), empathic concern (b), personal distress (c), perspective taking (d), and fantasy (e).

**Table S1**: Associations between overall empathy, cognitive and affective empathy subdomains, and burnout syndrome across the studies included in the meta‐analysis.

**Table S2**: Assessment of risk of bias of articles included in meta‐analysis.
