# Supplementary material for: Impact of Subdomains of Affective and Cognitive Empathy on Burnout Syndrome in Nurses: A Meta‐Analysis
Source: Int Nurs Rev. 2026 Mar 19;73(1):e70173. doi: 10.1111/inr.70173 (PMC13002559; doi:10.1111/inr.70173)
Supplement: Supplementary file 1 — Figure S1: Funnel plot assessing publication bias in the meta‐analyses of overall empathy (a), empathic concern (b), personal distress (c), perspective taking (d), and fantasy (e). [file INR-73-0-s001.docx]

**
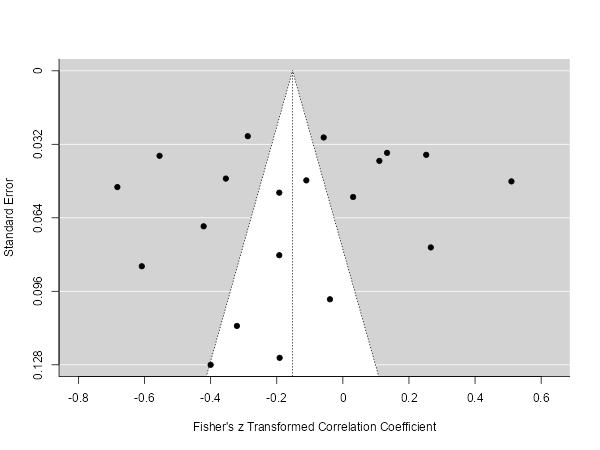
**

**Fig. a.**

**
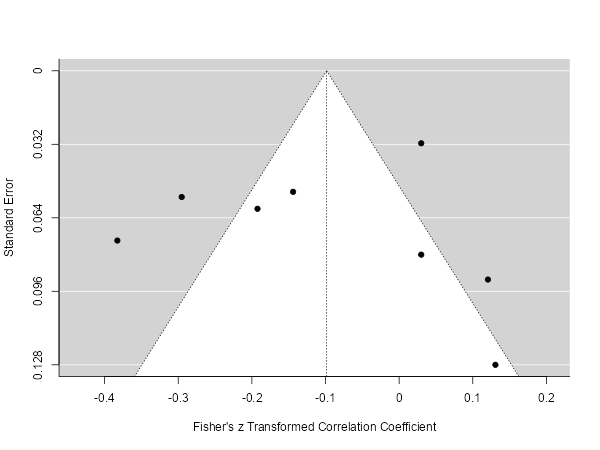
**
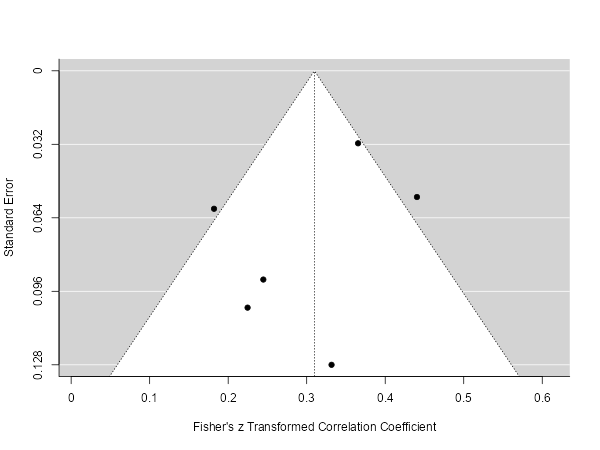


**Fig. b Fig. c**


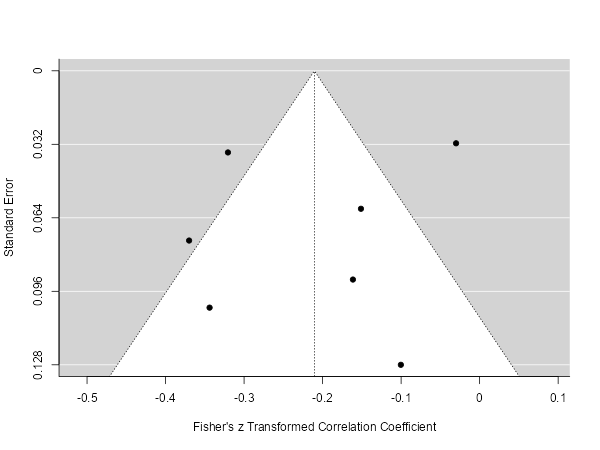
**
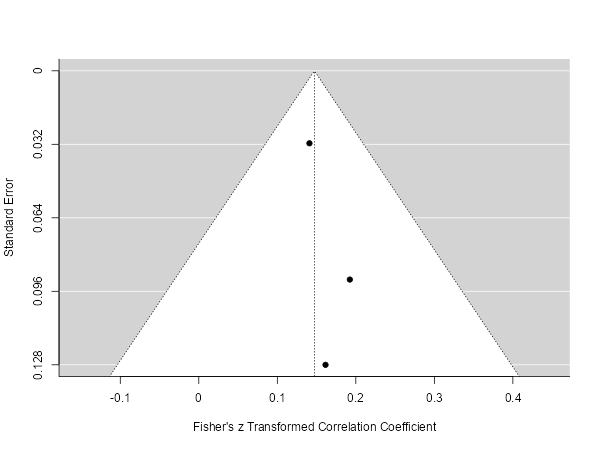
**

**Fig. d Fig. e**

**Supplementary Figure 1.** Funnel plot assessing publication bias in the meta-analyses of overall empathy (a), empathic concern (b), personal distress (c), perspective taking (d), and fantasy (e).
