# Supplementary material for: Impact of Subdomains of Affective and Cognitive Empathy on Burnout Syndrome in Nurses: A Meta‐Analysis
Source: Int Nurs Rev. 2026 Mar 19;73(1):e70173. doi: 10.1111/inr.70173 (PMC13002559; doi:10.1111/inr.70173)
Supplement: Supplementary file 2 — Table S1: Associations between overall empathy, cognitive and affective empathy subdomains, and burnout syndrome across the studies included in the meta‐analysis. [file INR-73-0-s002.docx]

**Supplementary Table 1.**

*Associations between overall empathy, cognitive and affective empathy subdomains, and burnout syndrome across the studies included in the meta-analysis.*

| **Reference** | **Outcome** | |
| --- | --- | --- |
| Altmann (2021) | ↑ Empathy vs. ↑ Personal burnout | (*r* = 0.26; p < 0.05) |
| Åström et al. (1990) | ↑ Empathy vs. ↓ Burnout | (*r* = −0.19; p < 0.05) |
| Cao et al. (2021) | ↑ Empathy vs. ↓ Burnout | (*r* = −0.593; p < 0.001) |
| Caro et al. (2017) | ↑ Empathy vs. ↓ Burnout | (*r* = −0.38; p = 0.01) |
| Cheng et al. (2020) | ↑ Emotional experience vs. ↓ EE | (*r* = −0.047; p > 0.05) |
| Dor et al. (2018) | ↑ Empathy vs. ↓ EE | (*r* = −0.34; p < 0.001) |
| Duarte et al. (2016) | ↑ Empathic concern vs. ↓ Burnout  ↑ Personal distress vs. Burnout  ↑ Perspective taking vs. ↓ Burnout | (*r* = -0.19; p < 0.01)  (*r* = 0.18; p < 0.01)  (*r* = −0.15; p < 0.01) |
| Fitzgerald−Yau et al. (2006) | ↓ Empathy vs. ↓ EE | (*r* = −0.11; p < 0.05) |
| Gountas & Gountas, (2015) | ^†^Empathic concern vs. ^†^EE | (*r* = 0.03; p > 0.05) |
| Hui et al. (2019) | ↑ Empathy vs. ↓ Burnout | (*r* = −0.504. p < 0.01) |
| Kayikci et al. (2025) | ↑ Empathy vs. ↓ Burnout | (*r* = −0.543. p < 0.001) |
| Kitano et al. (2023) | ^†^Empathic concern vs. ^†^EE  ↑ Personal distress vs. ↑ EE  ^†^Perspective taking vs. ^†^EE  ↑ Fantasy vs. ↑ EE | (*r* = 0.03; p > 0.05)  (*r* = 0.35; p < 0.05)  (*r* = −0.03; p > 0.05)  (*r* = 0.14; p < 0.05) |
| Mersin et al. (2024) | ↑ Empathy vs. ↑ Burnout | (*r* = 0.11; p < 0.05) |
| Narme, (2018) | ^†^Empathic concern vs. ^†^EE  ↑ Personal distress vs. ↑ EE  ↑ Fantasy vs. ↑ EE  ^†^Perspective taking vs. ^†^EE | (*r* = 0.12; p > 0.05)  (*r* = 0.24; p <0.01)  (*r* = 0.19; p < 0.05)  (*r* = −0.16; p > 0.05) |
| Pérez−Fuentes et al. (2019) | ↑ Empathy vs. ↓ Burnout | (*r* = −0.28; p < 0.001) |
| Raižiene et al. (2007) | ↑ Empathy vs. ↓ EE | (*r* = −0.19; p < 0.05) |
| Ren et al. (2020) | ↑ Empathy vs. ↑ EE | (*r* = 0.133; p < 0.001) |
| Román−Sánche et al. (2022) | ↑ Empathy vs. ↑ Burnout | (*r* = 0.247; p < 0.01) |
| Şahin et al. (2018) | ^†^Empathy vs. ^†^EE | (*r* = 0.031; p = 0.568) |
| Salvarani et al. (2019) | ↑ Personal distress vs. ↑ EE  ↑ Perspective taking vs. ↓ EE | (*r* = 0.221; p < 0.05)  (*r* = −0.331; p < 0.01) |
| Serrada-Tejeda et al. (2025) | ^†^Empathy vs. ^†^Burnout | (*r* = −0.039; p > 0.05) |
| Shi et al. (2022) | ↑ Personal distress vs. ↑ Burnout  ↑ Empathic concern vs. ↓ Burnout  ↑ Perspective taking vs. ↓ Burnout | (*r* = 0.414; p < 0.01)  (*r* = −0.287; p < 0.01)  (*r* = −0.310; p < 0.01) |
| Taleghani et al. (2017) | ↑ Empathy vs. ↓ Burnout | (*r* = −0.189; p = 0.04) |
| Topçu et al. (2023) | ↑ Empathy vs. ↑ Burnout | (*r* = 0.470; p < 0.01) |
| Wilczek−Ruzyczka, (2020) | ↑ Personal distress vs. ↑ EE  ↑ Empathic concern vs. ↑ EE  ^†^Perspective taking vs. ^†^EE  ^†^Fantasy vs. ^†^EE | (*r* = 0.32; p < 0.05)  (*r* = 0.13; p = 0.30)  (*r* = −0.10; p = 0.42)  (*r* = 0.16; p = 0.21) |
| Ye et al. (2024) | ↑ Empathy vs. ↓ Burnout | (*r* = −0.398; p < 0.001) |
| Yıldırım et al. (2024) | ^†^Empathy vs. ^†^Burnout | (*r* = −0.058; p > 0.05) |
| Yu et al. (2021) | ↑ Empathic concern vs. ↓ Burnout  ↑ Perspective taking vs. ↓ Burnout | (*r* = −0.365; p < 0.01)  (*r* = −0.354; p < 0.01) |
| Załuski et al. (2020) | ↑ Empathy vs. ↓ Burnout | (*r* = −0.31; p < 0.01) |

†: No significant difference. ↑: higher levels. ↓: lower levels. EE: emotional exhaustion.
