# Supplementary material for: Impact of Subdomains of Affective and Cognitive Empathy on Burnout Syndrome in Nurses: A Meta‐Analysis
Source: Int Nurs Rev. 2026 Mar 19;73(1):e70173. doi: 10.1111/inr.70173 (PMC13002559; doi:10.1111/inr.70173)
Supplement: Supplementary file 3 — Table S2: Assessment of risk of bias of articles included in meta‐analysis. [file INR-73-0-s003.docx]

**Supplementary Table 2**.

*Assessment of risk of bias of articles included in meta-analysis.*

| **References** | **Items** | | | | | | | | **Score total** | **Total (%)** |
| --- | --- | --- | --- | --- | --- | --- | --- | --- | --- | --- |
|  | **1** | **2** | **3** | **4** | **5** | **6** | **7** | **8** |  |  |
| Altmann (2021) | **+** | **+** | **+** | **+** | **−** | **−** | **+** | **+** | 6 | 75*.*0 |
| Åström et al. (1990) | **+** | **+** | **+** | **+** | **+** | **−** | **+** | **+** | 7 | 87*.*5 |
| Cao et al. (2021) | **+** | **+** | **+** | **+** | **+** | **+** | **+** | **+** | 8 | 100 |
| Caro et al. (2017) | **−** | **+** | **−** | **+** | **−** | **−** | **+** | **+** | 4 | 50*.*0 |
| Cheng et al. (2020) | **+** | **+** | **+** | **+** | **+** | **−** | **+** | **+** | 7 | 87*.*5 |
| Dor et al. (2018) | **+** | **+** | **+** | **+** | **+** | **+** | **+** | **+** | 8 | 100.0 |
| Duarte et al. (2016) | **+** | **+** | **+** | **+** | **−** | **−** | **+** | **+** | 6 | 75*.*0 |
| Fitzgerald−Yau et al. (2006) | **+** | **+** | **+** | **+** | **−** | **−** | **+** | **+** | 6 | 75*.*0 |
| Gountas & Gountas, (2015) | **+** | **+** | **+** | **+** | **−** | **−** | **+** | **+** | 6 | 75*.*0 |
| Hui et al. (2019) | **+** | **+** | **+** | **+** | **−** | **−** | **+** | **+** | 6 | 75*.*0 |
| Kayikci et al. (2025) | **−** | **−** | **+** | **+** | **+** | **−** | **+** | **+** | 5 | 62*.*5 |
| Kitano et al. (2023) | **−** | **+** | **+** | **+** | **+** | **+** | **+** | **+** | 7 | 87*.*5 |
| Mersin et al. (2024) | **+** | **+** | **+** | **+** | **−** | **−** | **+** | **+** | 6 | 75*.*0 |
| Narme, (2018) | **+** | **+** | **−** | **+** | **−** | **−** | **+** | **+** | 5 | 62*.*5 |
| Pérez−Fuentes et al. (2019) | **−** | **+** | **+** | **+** | **+** | **+** | **+** | **+** | 7 | 87*.*5 |
| Raižiene et al. (2007) | **−** | **−** | **+** | **+** | **+** | **−** | **+** | **+** | 5 | 62*.*5 |
| Ren et al. (2020) | **+** | **+** | **+** | **+** | **+** | **+** | **+** | **+** | 8 | 100.0 |
| Román−Sánche et al. (2022) | **+** | **+** | **+** | **+** | **+** | **+** | **+** | **+** | 8 | 100.0 |
| Şahin et al. (2018) | **+** | **+** | **+** | **+** | **+** | **+** | **+** | **+** | 8 | 100.0 |
| Salvarani et al. (2019) | **+** | **+** | **+** | **+** | **+** | **+** | **+** | **+** | 8 | 100.0 |
| Serrada-Tejeda et al. (2025) | **+** | **+** | **+** | **+** | **+** | **+** | **+** | **+** | 8 | 100.0 |
| Shi et al. (2022) | **+** | **+** | **+** | **+** | **+** | **−** | **+** | **+** | 7 | 87*.*5 |
| Taleghani et al. (2017) | **+** | **+** | **+** | **+** | **−** | **−** | **+** | **+** | 6 | 75*.*0 |
| Topçu et al. (2023) | **+** | **+** | **+** | **+** | **−** | **−** | **+** | **+** | 6 | 75*.*0 |
| Wilczek−Ruzyczka, (2020) | **+** | **+** | **+** | **+** | **−** | **−** | **+** | **+** | 6 | 75*.*0 |
| Ye et al. (2024) | **+** | **+** | **+** | **+** | **+** | **+** | **+** | **+** | 8 | 100.0 |
| Yıldırım et al. (2024) | **+** | **+** | **+** | **+** | **+** | **+** | **+** | **+** | 8 | 100.0 |
| Yu et al. (2021) | **+** | **+** | **+** | **+** | **+** | **+** | **+** | **+** | 8 | 100.0 |
| Załuski et al. (2020) | **+** | **+** | **+** | **+** | **+** | **?** | **+** | **+** | 7 | 87*.*5 |

1. Were the criteria or inclusion in the sample clearly defined? 2. Were the study subjects and setting described in detail? 3. Was the exposure measured in a valid and reliable way? 4. Were objective, standard criteria used or measurement of the condition? 5. Were confounding factors identified? 6. Were strategies to deal with confounding factors stated? 7. Were the outcomes measured in a valid and reliable way? 8. Was appropriate statistical analysis used? +:Yes. −: No. ?: Unclear.
